# Molecular Investigation of the Stem Snap Point in Textile Hemp

**DOI:** 10.3390/genes8120363

**Published:** 2017-12-04

**Authors:** Marc Behr, Sylvain Legay, Jean-Francois Hausman, Stanley Lutts, Gea Guerriero

**Affiliations:** 1 Environmental Research and Innovation Department, Luxembourg Institute of Science and Technology, 5 avenue des Hauts-Fourneaux, L-4362 Esch/Alzette, Luxembourg; marc.behr@list.lu (M.B.); sylvain.legay@list.lu (S.L.); jean-francois.hausman@list.lu (J.-F.H.); 2 Groupe de Recherche en Physiologie Végétale, Earth and Life Institute-Agronomy, Université catholique de Louvain, 5 (Bte 7.07.13) Place Croix du Sud, 1348 Louvain-la-Neuve, Belgium; stanley.lutts@uclouvain.be

**Keywords:** bast fibres, cell wall, gene expression, hemp, snap point

## Abstract

Fibre crops are important natural resources, as they sustainably provide bast fibres, an economically-valuable raw material used in the textile and biocomposite sectors. Among fibre crops, textile hemp (*Cannabis sativa* L.) is appreciated for its long and strong gelatinous bast fibres. The stem of fibre crops is a useful system for cell wall-oriented studies, because it shows a strong tissue polarity with a lignified inner core and a cellulosic hypolignified cortex, as well as a basipetal lignification gradient. Along the stem axis of fibre crops, a specific region, denoted snap point, marks the transition from elongation (above it) to fibre thickening (below it). After empirically determining the snap point by tilting the plant, we divided the stem segment containing it into three non-overlapping consecutive regions measuring 1 cm each, and carried out targeted RT-qPCR on cell wall-related genes separately, in outer and inner tissues. Different gene clusters can be observed, two of which are the major gene groups, i.e., one group with members expressed at higher levels in the inner tissues, and one group whose genes are more expressed in the cortex. The present results provide a molecular validation that the snap point is characterised by a gradient of events associated with the shift from fibre elongation to thickening.

## 1. Introduction

The stems of fibre crops, such as hemp and flax, are characterised by the occurrence of both cellulosic and woody fibres, localised at the cortex and core, respectively. The cellulosic fibres are long cells developing a thick, tertiary cell wall, which is rich in crystalline cellulose and similar to the gelatinous (G) layer forming in tension wood (TW) [1,2,3].

Along the stem axis of fibre crops, a gradient accompanying the development of bast fibres and vascular tissues is present; young internodes elongate rapidly, while at the base, older internodes cease elongation and thicken. Bast fibres elongate mostly by intrusive growth and undergo secondary and tertiary cell wall deposition only when elongation has stopped [4]. The shift to secondary and tertiary cell wall (also known as G-layer) deposition results in changes in the mechanical properties of the stem; a region physically marking the boundaries of this transition is easily recognizable by tilting the stem of fibre crops, and is referred to as the snap point [5,6]. In our previous transcriptomic study, we showed that the majority of secondary cell wall biosynthetic processes in the bast fibres take place at the snap point in textile hemp [7], where genes involved in both hemicellulose and lignin metabolism are significantly upregulated. Here, we have focused our attention on the snap point of textile hemp, and provided evidence for the existence of a gradient of cell wall-related processes in this crucial stem region. We performed a molecular dissection of the stem internode comprising the snap point region, by dividing it into three 1 cm non-overlapping consecutive disks. We separated the cortical and core tissues of each disk, and carried out targeted RT-qPCR. We monitored the expression of key genes involved in cellulose and lignin biosynthesis, previously identified as being highly dynamic in the hemp hypocotyl and stem [7,8,9]. Our results provide further insight into the regulation of cell wall-related genes in the stem tissues of textile hemp.

## 2. Materials and Methods

### 2.1. Plant Material and Growth Conditions

**Figure 1:**
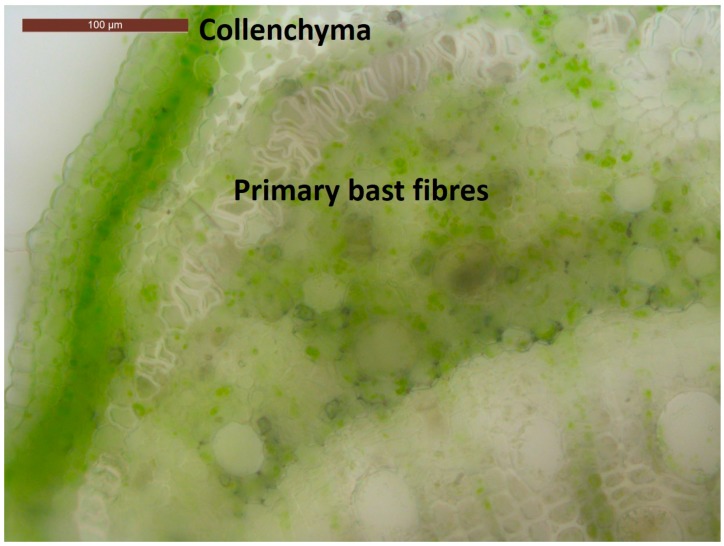
Cross-section of the snap point showing the thickening of primary bast fibres. Scale bar is 100 µm.

A monoecious hemp fibre variety (*Cannabis sativa* cv. Santhica 27) was analysed in this work (seeds certified by the Service Officiel de Contrôle et de Certification (SOC) of the Groupement National Interprofessionel des Semences et plants (GNIS), France). Plants were grown as previously described [10] and sampled at the age of six weeks (at a height of 100 cm–120 cm). The snap point was located 5.5 cm to 6.5 cm below the stem apex. Disks of 1 cm were excised from a 3 cm internode segment containing the snap point. SP1, SP2 and SP3 refer to the top, medium and basal disks of the excised segment, respectively. The snap point was determined by gently tilting the plant and by identifying the region of the stem showing a kink [6]. Subsequently, the stem region containing the kink was quickly excised (1.5 cm above and below the kink). The cortical tissues containing epidermal, parenchymatic and fibre cells (labelled OUT) were peeled from the shivs (labelled IN), and quickly frozen in liquid nitrogen. The number of biological replicates was four, with one plant in each replicate. A representative microscopic observation of the sampled region, prepared as described in [9], is depicted in Figure 1. 

### 2.2. RNA Extraction and RT-qPCR

Total RNA was extracted using the RNeasy Plant Mini Kit (Qiagen, Leusden, The Netherlands), coupled with on-column DNase treatment. The RNA concentration was measured with a Nanodrop ND-1000 (Thermo Scientific, Waltham, MA, USA). One microgram of total RNA was retrotranscribed into cDNA using the ProtoScript II Reverse transcriptase (New England Biolabs, Leiden, The Netherlands) and random primers oligonucleotides (Invitrogen, Carlsbad, CA, USA), according to the manufacturer’s instructions. The cDNA (4 ng) was used for the RT-qPCR analysis with Takyon SYBR Green low ROX (Eurogentec, Seraing, Belgium) for a total reaction volume of 10 µL in 384-wells microplates. An automated liquid handling robot (epMotion 5073, Eppendorf, Hamburg, Germany) was used to prepare the microplates. The expression of each target gene was normalised using two reference genes (*Cyclophilin* and *Etif3H*), selected among six (*Clathrin*, *F-Box*, *Etif3E* and *Ubiquitin*). The specificity of the amplicons was checked with a melt curve analysis. The normalised relative expression of the genes was calculated in qBasePLUS [11]. The primers used in this study have been previously described and validated [7,8,9,10], or designed based on the transcript sequences retrieved from the Medicinal Plant Genomics Resource database [12] (Table S1). The normalised relative expression values are indicated as a heat map hierarchical clustering, performed with the software PermutMatrix [13], using the following parameters: dissimilarity assessed by Pearson distance, clustering in complete linkage, seriation and tree seriation in multiple-fragment heuristic (MF), and rows normalised by Z-score scaling. The genes were named according to the *Arabidopsis thaliana* nomenclature. Two statistical tests have been performed: (i) an ANOVA or a Kruskall–Wallis on the whole set of samples (SP1, SP2 and SP3 IN and OUT), and (ii) a Student *t*-test between the different heights of the inner and outer tissues, to follow their individual developments (Table S1).

## 3. Results and Discussion

**Figure 2:**
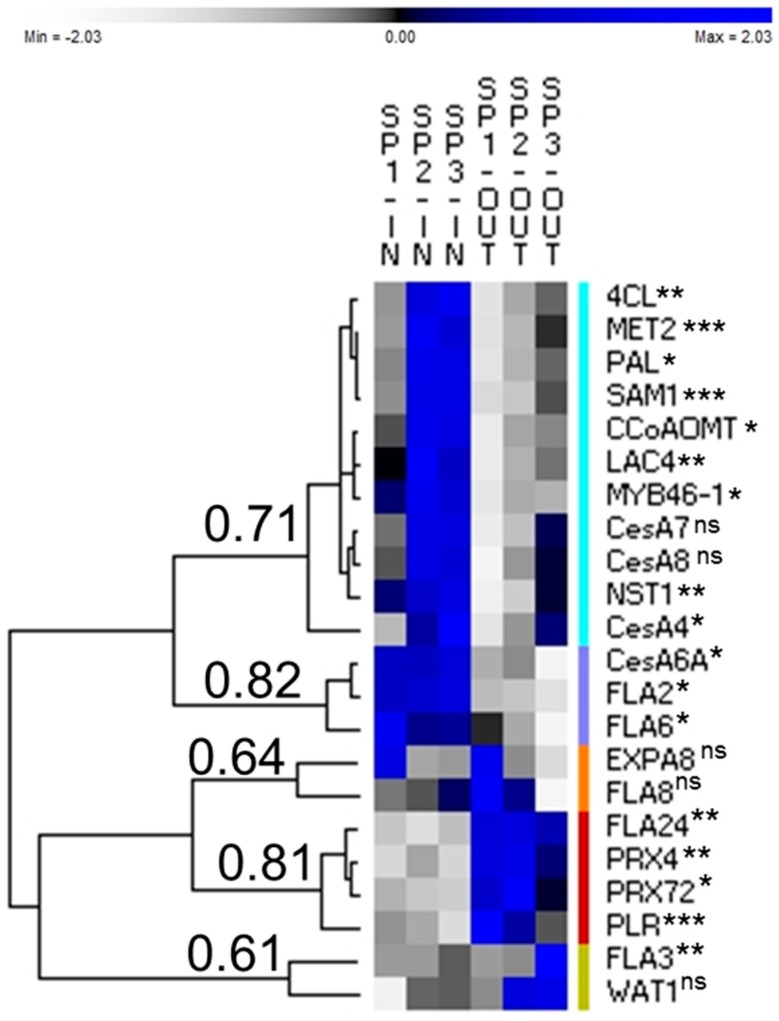
Normalised relative expression values of the six samples for each gene as calculated by qBASE^+^ and PermutMatrix. *N* = 4. First cluster in turquoise; second cluster in violet; third cluster in orange; fourth cluster in red and fifth cluster in dark yellow. *p*-value < 0.05 *, 0.01 **, 0.001 *** and > 0.05^ns^ from ANOVA or Kruskall–Wallis test (Table S1). The Pearson correlation coefficient is indicated for each group. The normalised relative expression values and statistical tests are in Table S1. Abbreviations as in the text.

A total of 22 cell wall-related genes, previously investigated in hemp [7,8,9], and related to cellulose [14], lignin [15] and cell wall organisation [9] were targeted using RT-qPCR. The heat map hierarchical clustering of the normalised relative expressions indicates the presence of five major groups (Figure 2). The first group is composed of eleven genes belonging to the secondary cell wall (SCW) and lignin biosynthetic process, and shows increased expression in the inner tissues (with upregulation in SP2 and SP3). The following genes are found in this cluster: *4-coumarate CoA ligase* (*4CL*), *Methionine synthase 2* (*MET2*), *Phenylalanine ammonia-lyase* (*PAL*), *S-adenosylmethionine synthetase 1* (*SAM1*), *Caffeoyl-CoA 3-O-methyltransferase* (*CCoAOMT*), *Laccase 4* (*LAC4*), *MYB46-1*, *Cellulose synthase 7* (*CesA7*), *CesA8*, *NAC secondary cell wall thickening 1* (*NST1*) and *CesA4*. *CesA4* was slightly less expressed than the other genes of this cluster in SP1-IN, explaining its specific position in this group. The second group shows higher expression in the core tissue of the different disks, with respect to the cortex (within the cortex, there was a gradual decrease in expression from SP1 to SP3). This group gathers the primary cell wall (PCW)-related genes *CesA6A*, *Fasciclin-like arabinogalactan 2* (*FLA2*) and *FLA6*.

Genes clustered in the third group are the elongation-related genes *α-expansin 8* (*EXPA8*) and *FLA8*. Their expression is high in the cortex of SP1 and lower in the cortical tissues of SP2 and SP3. The genes of the fourth group *Peroxidase 4* (*PRX4*), *PRX72*, and *Pinoresinol lariciresinol reductase* (*PLR*) show upregulation in the cortex of SP1 and SP2. Finally, the expression of the genes of the fifth group *FLA3* and *Walls are thin 1* (*WAT1*) increases in the cortex of SP2 and SP3.

### 3.1. Genes Preferentially Expressed in the Inner Tissues

The genes of the first group belong to the SCW-biosynthetic program. Their expressions are regulated by the two transcription factors (TFs) NST1 and MYB46-1. NST1 is the master regulator of fibre differentiation [15] and MYB46-1 is a tier 2 transcription factor involved in SCW biogenesis and xylan biosynthesis [16]. Therefore, it is not surprising that the genes involved in cellulose deposition in the SCW (*CesA4*, *CesA7* and *CesA8*) and in monolignol biosynthesis (*PAL*, *4CL*, *CCoAOMT* and the methyl donors *SAM1* and *MET2*) display a similar expression pattern. These genes are more expressed in the inner tissue of the lower part stem, containing the xylem cells with xylan-type SCW. These data are in agreement with the results of van den Broeck and colleagues [17], which show in the core tissue an upregulation of *PAL*, *4CL* and *CCoAOMT* for monolignol biosynthesis, and *SAM* and *MET* for the methyl donor metabolism. Hemp bast fibres are weakly lignified, but nevertheless, we observed a significantly increased expression of the genes belonging to the first group in the basal segment (SP3-OUT), as compared to SP1-OUT (Table S1). *PAL1* and *Caffeic acid O-methyltransferase* (*COMT*) were also significantly more expressed in the bast fibres located below the snap point of hemp stem [7]. Methylation of monolignols is catalysed by COMT and CCoAOMT and *S*-adenosylmethionine are methyl donors for this reaction, explaining the higher transcription of genes related to this metabolism, such as *SAM1* and *MET2* in the core tissue. The expression of *CesA4*, *CesA7* and *CesA8* slightly increases in SP2 and SP3, compared to SP1, both in the core and the cortex. *CesA8* shows a significant upregulation at SP3-OUT (fold change SP3-OUT vs. SP2-OUT and SP1-OUT of 1.3 and 4.4, respectively, Table S1). The onset of bast fibre SCW deposition occurs at the snap point. Because of their vicinity, the histology of the three consecutive segments is very similar, but our sampling strategy enabled the detection of differences, even if they may be small, in the expression of genes related to SCW deposition. This trend was strong when observed in different internodes; the fold change of *CesA8* between the internode of the snap point and the internode above the snap point was around 11 [7].

*CesA6A*, *FLA2* and *FLA6* are the genes of the second group (Figure 2). These genes are more expressed in the core as compared to the cortex (Table S1). In the outer tissue, *FLA6* is significantly more expressed in SP1 than in SP3 (Table S1). CESA6 is involved in the deposition of cellulose in the PCW [18], but is also present in flax fibres depositing the G-layer [19]. CESA6 has different isoforms (5 in flax according to [20]). Additional characterisations of hemp *CesA* genes are required to define the exact number of isoforms for each *CesA*, and to clearly define their respective expression patterns in the different tissues/stem regions. *FLA2* and *FLA6* are more expressed in the core and cortical tissues of younger internodes [9]. The closest ortholog of hemp CsaFLA6 in *Arabidopsis* is FLA9 [9]. Expression of *AtFLA9* is significantly repressed in a mutant with altered auxin signalling, caused by loss of function of several *VIER F-BOX PROTEINES* (*VFB*s). Loss of *VFB* function by RNA interference causes changes in gene expression, and delays plant growth [21]. The *FLA9* expression level is also negatively affected in the *Arabidopsis* auxin-insensitive gain-of-function mutant *iaa3* but is higher in seedlings exposed to brassinolide, a brassinosteroid [22]. Interestingly, the expression of *EXPA8* is also induced by auxin [23], and brassinolide [22], showing a pattern similar to *CsaFLA6* (Figure 2). Considering the significantly higher expression of *CsaFLA6* and *EXPA8* in outer SP1 compared to SP3 (Table S1), we may speculate that these proteins play a role in the elongation of the bast fibres, in relation with auxin and brassinosteroids.

### 3.2. Genes Preferentially Expressed in the Outer Tissues

Expression of the genes of the third cluster, *EXPA8* and *FLA8*, does not change significantly across the disk sections according to the ANOVA test (Table S1). However, the expression of *EXPA8* decreases from the top to the bottom, both in inner and outer tissues, resulting in a significant difference between SP1-OUT and SP3-OUT. Expansins are involved in cell elongation, mediating the degradation of the biomechanical hotspots where xyloglucan and cellulose are closely intertwined [24]. In this respect, the expression of *EXPA8* was higher in elongating hemp hypocotyls, as compared to thickening hypocotyls [8], and the bast fibres of younger internodes [7]. Using a microarrays approach, a transcript for a putative expansin was upregulated in the bast fibres of the upper part of hemp stem [25]. Therefore, one may suppose that the expression of *EXPA8* is not drastically changing because stem elongation around the snap point is very limited, as compared to the stem part closer to the shoot apical meristem. Similarly, the relatively constant expression of *CsaFLA8*, which was shown to be upregulated during bast fibre elongation [9], may be due to the limited elongation in the sampled region.

The genes of the fourth group are more expressed in the outer tissue. The *PLR* transcript abundance significantly decreases from the top to the bottom in both tissues, similarly to what was observed previously [7]. PLR is involved in the biosynthesis of lignans, such as pinoresinol. Lignans are monolignol-derived molecules, which are highly accumulated in the hypolignified bast fibres of flax [26], and may thus be final acceptors of the products of the monolignol pathway in hemp bast fibre. Stem and root elongation is regulated by specific lignans, such as syringaresinol and sesamin, in lettuce and ryegrass [27]. We can thus suggest that lignans may partake in organ elongation in hemp. Interestingly, the hemp genes annotated as *PRX4* and *PRX72* share a similar expression pattern with *PLR*. Alignment of hemp and thale cress protein sequences of PRX4 and PRX72 with the EMBOSS Needle suite [28] has shown identity/similarity rates of 62.8%/78.0% for PRX4 and 77.2%/87.8% for PRX72. AtPRX5 and AtPRX72 are the closest orthologs of CsaPRX4 and CsaPRX72, respectively, according to BLASTP [29] (*E* values of 3.10^−149^ and 0, respectively). In *Arabidopsis*, PRX4 and PRX72 are involved in the formation of syringyl lignin [30,31]. Considering that these two genes are more expressed in the outer tissue, which is poor in lignin and follow an acropetal gradient of expression, we suggest that, in hemp, they are not involved in lignin polymerisation, and that they are not functional orthologs of AtPRX4 and AtPRX72. They may be involved in other cellular processes occurring in the apoplast (e.g., protein bridges). In *FLA24*, the last gene of this cluster, no significant change in its expression was observed, except that it was slightly more expressed in the cortical tissue.

The last cluster is composed of *FLA3* and *WAT1*. Both genes are more expressed in the outer tissue and in the section undergoing SCW deposition (SP3). The expression of *FLA3* is significantly higher in SP3 than in SP1 and SP2, in both inner and outer tissues (Table S1). This result confirms the data obtained (i) with the time-course analysis of the hemp hypocotyl, where *CsaFLA3* was more expressed in hypocotyl undergoing secondary growth [8], and (ii) with hemp internodes at different developmental stages, where the expression of *CsaFLA3* peaked in the internode below the snap point [9]. Hemp FLA3 shows a single-fasciclin (FAS) domain, an N-terminal signal peptide and a C-terminal glycosylphosphatidylinositol (GPI) membrane anchor at the C-terminus mediating attachment to the cell surface and is closely related to AtFLA11 (thale cress), EgrFLA2 (*Eucalyptus grandis*) and PtrFLA6 (*Populus trichocarpa*) [9]. Importantly, the transcripts of several *FLA* are more abundant during the formation of TW in poplar [32]. Recently, it was demonstrated in poplars that gibberellins positively regulate TW formation by inducing the expression of several *FLA*s including *PtrFLA6* [33]. It has been shown in trees and thale cress that the expression of single-FAS domain *FLA* correlates with cellulose microfibril angle (MFA) and thus, wood stiffness [34]. In this respect, eucalypt *35S*:FLA2 fibres have a reduced MFA and increased stiffness and cellulose crystallinity. In *Arabidopsis*, the *fla11* insertion line shows a subtle irregular xylem phenotype (IRX13), as well as alterations in non-cellulosic polymers and a cellulose reduction, which may be linked to *FLA11* strong coexpression with *CesA4*, *CesA7* and *CesA8* [14]. Therefore, we strengthen the hypothesis that CsaFLA3 may be involved in SCW and G-layer deposition during the thickening stage. Functional analyses of *CsaFLA3*, or closely related *FLA* genes in other fibre crops, such as flax, may shed light on their roles in G-layer assembly. The expression of *WAT1* is slightly higher at the bottom part of the snap point internode, both in the inner and outer tissue (Table S1). WAT1 is a vacuolar auxin efflux transporter playing a key role in SCW thickening of fibres [35]. The *wat1* mutant shows altered expression in several transcription factors (*NST3*, *MYB46* and *MYB85*) and structural genes (*CesA4*, *CesA7*, *CesA8* and *IRX9*) important for SCW development [36,37]. The authors also showed that the early differentiation of fibres was not impacted in the *wat1* mutant, contrasting with later stage of SCW deposition, when the monolignol profile of the mutant is significantly altered. A role in fibre lignification is also possible, as the *wat1* mutant shows a lower amount of mono/oligolignols, but higher quantity of lignans [37]. It is therefore plausible that the gene expression of the *WAT1* hemp ortholog does not change dramatically in our experimental set-up because the fibres are not yet in their late stage of development. In support of this hypothesis, *WAT1* was found to be significantly more expressed in the internode below the snap point, as compared to the internode above the snap point [7].

## 4. Conclusions

In this study, we have characterised, by a gene expression analysis, some molecular changes occurring at the snap point in textile hemp. The expression profile of genes known to be involved in elongation (*expansin* or specific *FLA*s) and SCW deposition (*CesA*s, lignin biosynthesis) highlights the shift from fibre elongation to fibre thickening. Outer and inner tissues have contrasting patterns of gene expression, especially with genes involved in the monolignol/lignin biosynthetic pathway. It is also noteworthy to observe that some *FLA* genes, whose roles are just starting to be understood in woody and herbaceous species, are more expressed in elongating tissues (e.g., *FLA6*), while others are upregulated in tissues undergoing SCW deposition (e.g., *FLA3*). It will be interesting to study how the expression of the genes involved in the transition from elongation to thickening at the snap point, differs in similar fibre crops, such as flax or ramie, but also crops with xylan-type bast fibres, such as jute and kenaf. *FLA*s may play an important role, namely, concerning the different composition of the gelatinous-type and xylan-type cell wall. Such comparisons may help decipher the functions of some genes whose roles are still enigmatic.

## Supplementary Materials

The following are available online at www.mdpi.com/2073-4425/8/12/363/s1. Table S1: Primers and raw gene expression data from Figure 2.
